# Induction of Thioredoxin Reductase 1 by Korean Red Ginseng Water Extract Regulates Cytoprotective Effects on Human Endothelial Cells

**DOI:** 10.1155/2015/972040

**Published:** 2015-07-08

**Authors:** Hye Rim Park, Seung Eun Lee, Hana Yang, Gun Woo Son, Young-Ho Jin, Yong Seek Park

**Affiliations:** ^1^Department of Microbiology, School of Medicine, Kyung Hee University, Seoul 130-701, Republic of Korea; ^2^Department of Physiology, School of Medicine, Kyung Hee University, Seoul 130-701, Republic of Korea

## Abstract

Korean Red Ginseng is a popular herbal medicine and is widely used in many food products. KRG has biological benefits related to vascular diseases including diabetes, hypertension, atherosclerosis, and other cardiac diseases and KRG has antioxidant and anti-hyperlipidemic actions. KRG decreases the level of oxidative stress and suppresses proinflammatory cytokines and cell adhesion molecules, thus protecting endothelial dysfunction. Mammalian Thioredoxin reductase 1 is an NADPH-dependent selenoprotein, essential for antioxidant defense and DNA synthesis and repair, that regulates the redox system by modulating redox-sensitive transcription factors and thiol-containing proteins. Here, we show that KRG water extract increases the expression of TrxR1 in human umbilical vein endothelial cells via the p38 and PKC-*δ* signaling pathways. The induction of TrxR1 expression by KRG was confirmed by Western blot analysis and reverse transcription polymerase chain reaction. However, the increase in TrxR1 expression was abolished by specific silencing of the p38 and PKC-*δ* genes. In addition, we demonstrated that auranofin, a TrxR1 inhibitor, weakens the protective effect of KRG against H_2_O_2_-induced cell death as measured by the terminal transferase dUTP nick end labeling assay. These results suggest that KRG may have protective effects in vascular diseases by upregulating TrxR1 in endothelial cells, thereby inhibiting the generation of reactive oxygen species and cell death.

## 1. Introduction

The vascular endothelium is composed of a monolayer of cells and is an important organ for controlling vascular functions [1, 2]. The endothelium has multiple functions including regulation of vascular growth and remodeling, modulation of immune and thrombotic responses, and control of homeostasis and angiogenesis through interactions between the vessel wall and the immune cells [3, 4]. Impairment of endothelial function is implicated in most cardiovascular diseases, including diabetes, atherosclerosis, coronary artery disease, and hypertension [5, 6]. Oxidative stress-generated reactive oxygen species (ROS) are a major cause of endothelial dysfunction and damage endothelial cells by increasing the antioxidant defense mechanisms, altering vascular integrity, and increasing the accumulation of lipids, and protein peroxidation [7–9]. These alterations in the structure and function of vessel cells promote vascular disease [10].

Korean Red Ginseng (KRG) is a traditional herbal medicine. Its consumption has recently been increasing owing to its inclusion in many food products, such as candy, snacks, jellies, and beverages [11]. KRG has many pharmacological and physiological protective benefits in various biological systems, including anti-inflammatory, antioxidant, anticancer, and antidiabetic effects [12, 13]. Recently, a number of studies have demonstrated the vascular protective properties of KRG including vasorelaxing and hypotensive effects [13, 14]. KRG increases the levels of nitric oxide (NO) and endothelial NO synthase (eNOS), which prevent endothelial cell damage and dysfunction [12, 15]. In addition, KRG protects endothelial cells by abrogating the production of NADPH-driven superoxide [14, 16], increases angiogenesis by signaling pathway activation, and improves endothelial function [17]. These protective effects of KRG may be beneficial in cardiovascular diseases including diabetes, hypertension, and atherosclerosis [18, 19].

Thioredoxin reductases (TrxR) are a family of selenocysteine-containing oxidoreductases that restore the reduced state of oxidized Thioredoxin (Trx) using electrons from NADPH + H^+^ [20]. There are three mammalian isoforms of TrxR: cytosolic TrxR1, mitochondrial TrxR2, and a testes-specific TrxR3 [21]. TrxR1 is well known as an antioxidant enzyme that modulates cellular function such as antiapoptosis, cell growth, and anti-inflammation [22, 23]. Thioredoxin systems, comprising Trx, TrxR, and NADPH, are highly conserved and play an essential role in redox regulation of immunomodulation, apoptosis, DNA synthesis, and cytotoxicant-induced oxidative stress [24–26]. Oxidized Trx regenerates other antioxidant enzymes such as peroxiredoxin and influences transcription factors including Fos, Jun, and p53, which decrease the levels of ROS [27]. Recent research findings suggest that suppression of the Thioredoxin system may cause oxidative stress and promote apoptotic cell death [21, 28].

In this study, we examined the role of TrxR1 in the vascular protective effects of KRG on endothelial cells. We demonstrated that KRG water extract upregulated the expression of TrxR1 in human umbilical vein endothelial cells (HUVECs). We have also investigated the involvement of ROS, p38, and PKC-*δ* signaling pathways, in the induction of KRG-stimulated TrxR1 in HUVECs.

## 2. Materials and Methods

### 2.1. Materials

Korean Red Ginseng powder was supplied by the Korea Ginseng and Tobacco Central Research Institute (Daejeon, Korea). M199 medium and fetal bovine serum were obtained from Welgene Inc. (Daegu, Korea). TRIzol reagent and Lipofectamine RNAiMAX were obtained from Invitrogen (Carlsbad, CA). Auranofin was purchased from Sigma Chemical (St. Louis, MO). Anti-Thioredoxin reductase 1 and anti-GAPDH were purchased from AbFrontier (Seoul, Korea). SB203580 and Rottlerin were supplied by Calbiochem (La Jolla, CA), p38 siRNA (#6564) was obtained from Cell Signaling Technology (Beverly, MA), and PKC-*δ* siRNA (SC-36253) was purchased from Santa Cruz Biotechnology (Santa Cruz, CA). All other chemicals and reagents were of analytical grade.

### 2.2. Preparation of Red Ginseng Water Extract

For preparation of Red Ginseng water extract, we adapted a method used in a previous study [29]. Korean Red Ginseng powder was soaked in water (1 : 25, w : w) for 3 h and then boiled for 40 min. After centrifugation at 3,000 rpm for 60 min, the supernatants of ginseng extract were further centrifuged at 13,000 rpm for 30 min and lyophilized. The resultant ginseng extracts were dissolved in pure water immediately prior to the experiment.

### 2.3. Cell Culture

HUVECs were maintained in M199 medium supplemented with 10% fetal bovine serum, 1% penicillin and streptomycin, 10 ng/mL human fibroblast growth factor, and 18 mU/mL heparin. The cells were grown at 37°C in a 5% CO_2_ atmosphere. HUVECs were grown to approximately 80% confluence, maintained with fresh medium as described above, and subcultured every 2 to 3 d. Cells within passages 4 to 9 were used for the experiments described here.

### 2.4. Western Blot Analysis

Cells were lysed in lysis buffer containing 1 mM PMSF, 2 *μ*g/mL leupeptin, 5 *μ*g/mL aprotinin, and 1 mM EDTA. We applied 20 *μ*g of the whole cell lysate proteins to each lane and analyzed them with Western blot. Western blot analysis was performed using monoclonal antibodies against mouse TrxR1 and monoclonal antibody against rabbit GAPDH. Horseradish peroxidase-conjugated anti-IgG antibodies were used as the secondary antibody to detect the relevant protein bands by enhanced chemiluminescence WESTSAVE-Up TM (AbFrontier, Seoul, Korea).

### 2.5. RNA Isolation and Reverse Transcriptase-Polymerase Chain Reaction

Cells were seeded in a 100 mm diameter plate containing M199 medium. After 24 h, KRG was added to a final concentration of 1 mg/mL and the cells were incubated for 18 h. RNA extraction was performed using 1 mL TRIzol reagent. The RNA pellets were washed in 70% ethanol, dried completely, and dissolved in diethylpyrocarbonate treated water to inhibit RNase. Total RNA was quantified using a ND-100 spectrometer (NanoDrop Technologies, Wilmington, DE). Polymerase chain reaction was performed using the synthesized cDNA as a template and primers specific for TrxR1, or *β*-actin, as a loading control. The primer sequences for human TrxR1 were 5′-GAAGATCTTCCCAAGTCCTATGAC-3′ (forward) and 5′-ATTTGTTGCCTTAATCCTGTGAGG-3′ (reverse) [30]. The amplified products were resolved by 1% agarose gel electrophoresis, stained with ethidium bromide, and photographed under ultraviolet light.

### 2.6. p38 and PKC-*δ* Silencing by siRNA

Cells were seeded in 6-well plates at a density of 2 × 10^5^ cells/well and transfected with specific or scrambled siRNA for 18 h. For each transfection, 500 *μ*L of transfection medium was added to 0.25–1 *μ*g or 10–30 nM of the siRNA duplex/transfection reagent mix (Lipofectamine RNAiMAX), and the entire volume was added gently to the cells.

### 2.7. Measurement of Promoter Activity

EpRE/ARE-luciferase (EpRE/ARE-Luc) reporter plasmid was a generous gift from Dr. Park, R. K. (Wonkwang University, Korea). EpRE/ARE-Luc was generated by transfer of the enhancer 2 (E2) and minimal promoter (MP) sequences into the luciferase reporter plasmid pGL3-Basic [31]. HEK 293 cells were transfected with an ARE-luciferase (ARE-Luc) reporter plasmid. For transfection with the reporter plasmid, cells were seeded in 24-well plates at a density of 1.0 × 10^5^ cells/well 1 day before transfection. A total of 450 ng of plasmid DNA, including the luciferase reporter and 200 ng of pcDNA3-*β*-gal, were transfected into cells using ExGenTM 500 reagent (Fermentas, Hanover, MD). After 24 h of transfection, cells were exposed for different times to various concentrations of KRG. Cells were then washed twice with PBS and lysed using reporter lysis buffer (Promega, Madison, WI). A luciferase assay system (Promega) was used on an aliquot of supernatant, according to the manufacturer's instructions, to measure the luciferase activity using a luminometer (Biolumat LB 9509, Berthold, Berlin, Germany). Induction was calculated from the intensity value from each experimental group divided by the value from the control group after normalization of transfection efficiency by the *β*-galactosidase assay (number 75705, Pierce, Rockford, IL, USA).

### 2.8. Measurement of Intracellular Reactive Oxygen Species Generation

Intracellular ROS in H_2_O_2_ treated HUVECs was examined using DCF/DA staining. HUVECs were seeded at a density of 4 × 10^5^ cells in 60 mm dishes. After 18 h of incubation with KRG in the presence or the absence of 2 *μ*M auranofin, cells were stained with 10 *μ*M DCF/DA for 55 min and then treated with 500 *μ*M H_2_O_2_ for 5 min. After washing with phosphate buffered saline (PBS), the cells were examined by fluorescence microscopy [32, 33].

### 2.9. Terminal Transferase dUTP Nick End Labeling Assay

Cells were seeded in 8-well chamber slides at a density of 2 × 10^4^ cells/300 *μ*L/well, rinsed with PBS, fixed in 4% formaldehyde for 1 h, and then exposed to a permeabilizing solution (0.1% Triton X-100 in 0.1% sodium citrate buffer) for 2 min at 4°C. Cells were then incubated with the terminal transferase dUTP nick end labeling (TUNEL) reaction mixture (Roche, Mannheim, Germany) for 1 h at 37°C in the dark. After washing with PBS, the cells were mounted and analyzed by fluorescence microscopy [34].

### 2.10. Statistical Analysis

Data were analyzed by Student's *t*-test and the results are expressed as mean ± S.D.

## 3. Results

### 3.1. Increase of TrxR1 in Human Umbilical Vein Endothelial Cells Treated with Korean Red Ginseng

**Figure 1:**
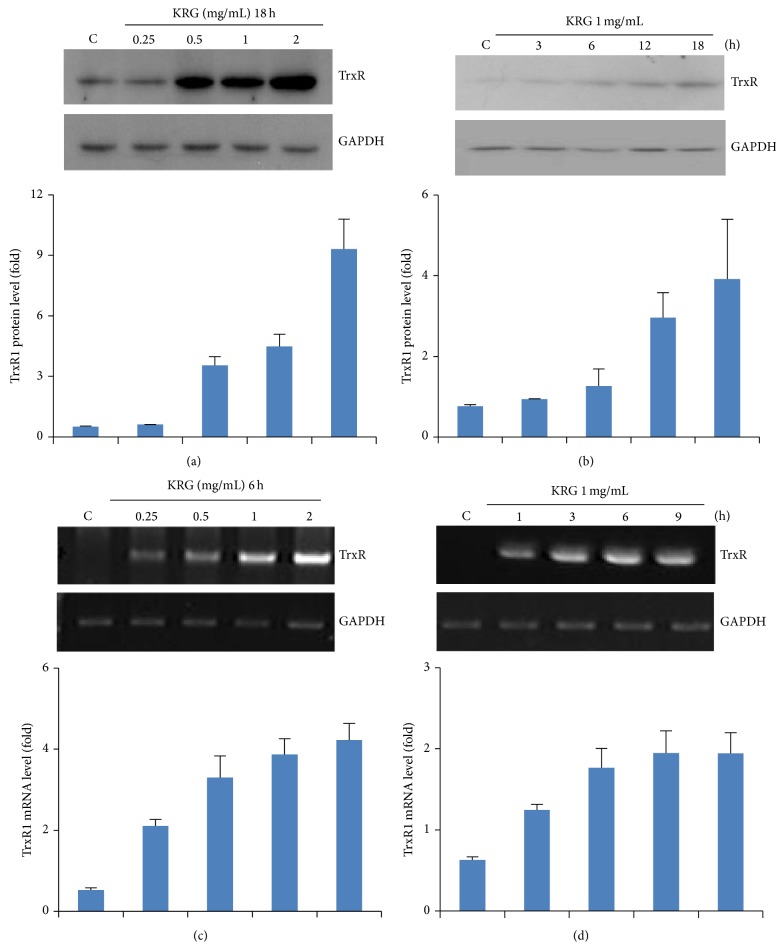
Effect of KRG on TrxR1 expression in HUVECs. Cells were treated with the indicated concentrations of KRG for 18 h and then the protein in cell lysates was analyzed by Western blot using TrxR1 specific antibody (a). RT-PCR was performed to measure the levels of TrxR1 mRNA transcript (c). Cells treated with 1 mg/mL KRG were harvested at various time intervals for analysis (b and d). The level of glyceraldehyde-3-phosphate dehydrogenase (GAPDH) was used as a loading control.

TrxR1 is an important antioxidant enzyme that regulates redox homeostasis. We first showed the effect of KRG on TrxR1 protein induction in various concentrations. Incubation of HUVECs with KRG (0–2 mg/L) for 18 h induced TrxR1 in a concentration-dependent manner (Figure 1(a)). Treatment of HUVECs with KRG at 1 mg/mL significantly increased the expression of TrxR1 protein in a time-dependent manner (Figure 1(b)). KRG also increased TrxR1 mRNA levels when cells were incubated with various concentrations of KRG (0–2 mg/L) for 6 h (Figure 1(c)), and this accumulation occurred in a time-dependent manner (Figure 1(d)).

### 3.2. TrxR1 Upregulation by KRG Is Mediated by the p38 Mitogen-Activated Protein Kinase (MAPK) and Protein Kinase C- (PKC-) *δ* Pathways

**Figure 2:**
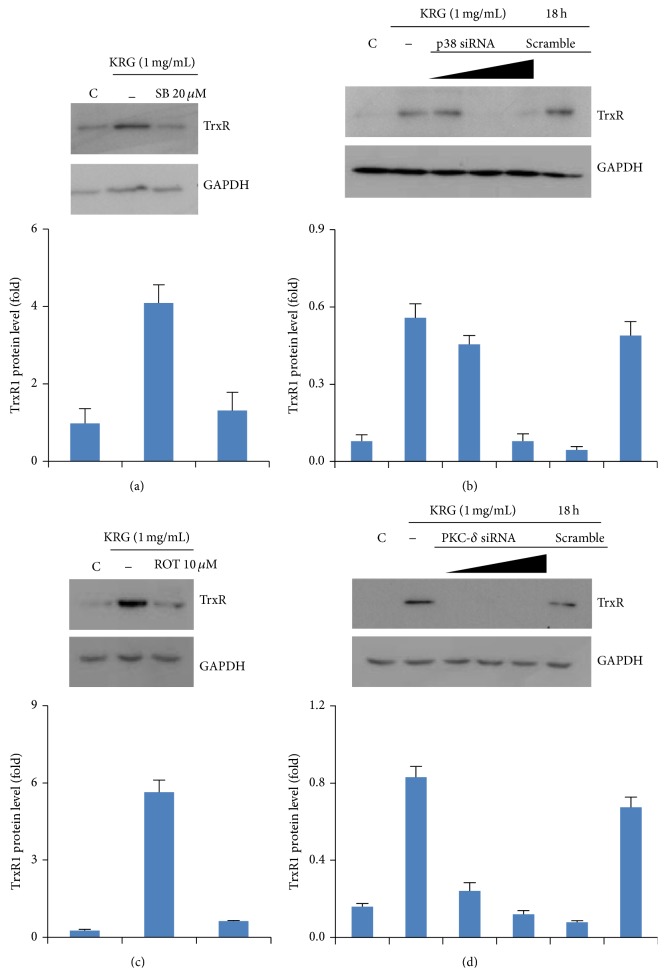
Involvement of p38 and PKC-*δ* signaling pathway in KRG-treated TrxR1 expression in HUVECs. Cells were pretreated with 20 *μ*M SB203580 (p38 inhibitor) or 10 *μ*M Rottlerin (PKC-*δ* inhibitor) for 1 h, followed by incubation with 1 mg/mL KRG for 18 h. Whole cell lysates were analyzed by Western blot with antibodies against TrxR1 and GAPDH (a and c). Transient transfection of cells with 10–30 nM p38 and PKC-*δ* siRNA suppressed the upregulation of TrxR1 expression by KRG (b and d).

TrxR1 expression is associated with a number of signaling pathways. To examine the upstream signaling pathway related to KRG-induced TrxR1, cells were pretreated with specific inhibitors of p38 MAPK (SB203580) or PKC-*δ* (Rottlerin) for 1 h before incubation with 1 mg/mL KRG for 18 h. Inhibitors of the p38 and PKC-*δ* pathways suppressed TrxR1 induction in KRG-treated HUVECs (Figures 2(a) and 2(c)). To confirm these results, we used specific small interfering RNAs (siRNAs) for p38 and PKC-*δ*. KRG-induced TrxR1 expression was abolished by transfection of either p38 or PKC-*δ* siRNA (Figures 2(b) and 2(d)). These results show that both the p38 and PKC-*δ* signaling pathways are associated with KRG-stimulated expression of TrxR1.

### 3.3. TrxR1 Induction by KRG Is Mediated by EpRE/ARE

**Figure 3:**
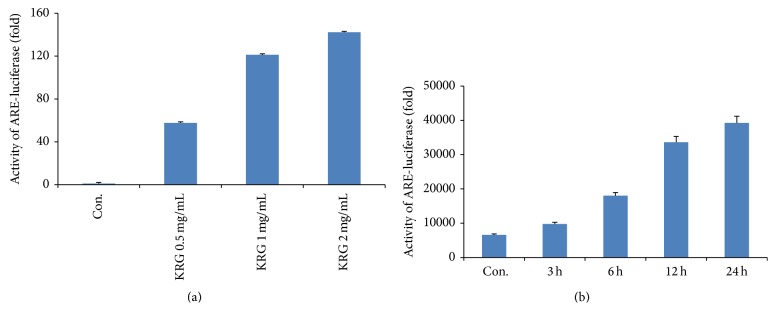
KRG-treated expression of TrxR1 is mediated by EpRE/ARE. Cells transfected with an EpRE/ARE-luciferase construct were stimulated with various concentrations of KRG for 6 h (a) and with 1 mg/mL of KRG for various time intervals (b). The lysates were mixed with a luciferase substrate and a luminometer was used to measure luciferase activity. Data represent the mean ± S.D-values of 3 independent experiments.

The electrophile/antioxidant response element (EpRE/ARE) is an important promoter region for antioxidant enzymes. To determine whether the transcriptional activity of EpRE/ARE is associated with KRG-stimulated TrxR1 induction, cells were transiently transfected with an EpRE/ARE-luciferase reporter plasmid and stimulated with different concentrations of KRG for 6 h and with 1 mg/mL KRG for various times. Luciferase activity was then measured. Cells transfected with the EpRE/ARE vector to KRG showed increased EpRE/ARE-luciferase activity (Figures 3(a) and 3(b)). This result suggests that EpRE/ARE plays an important role in the KRG-induced increase in TrxR1 expression.

### 3.4. Protection against Oxidative Stress Provided by TrxR1 Stimulated by KRG

**Figure 4:**
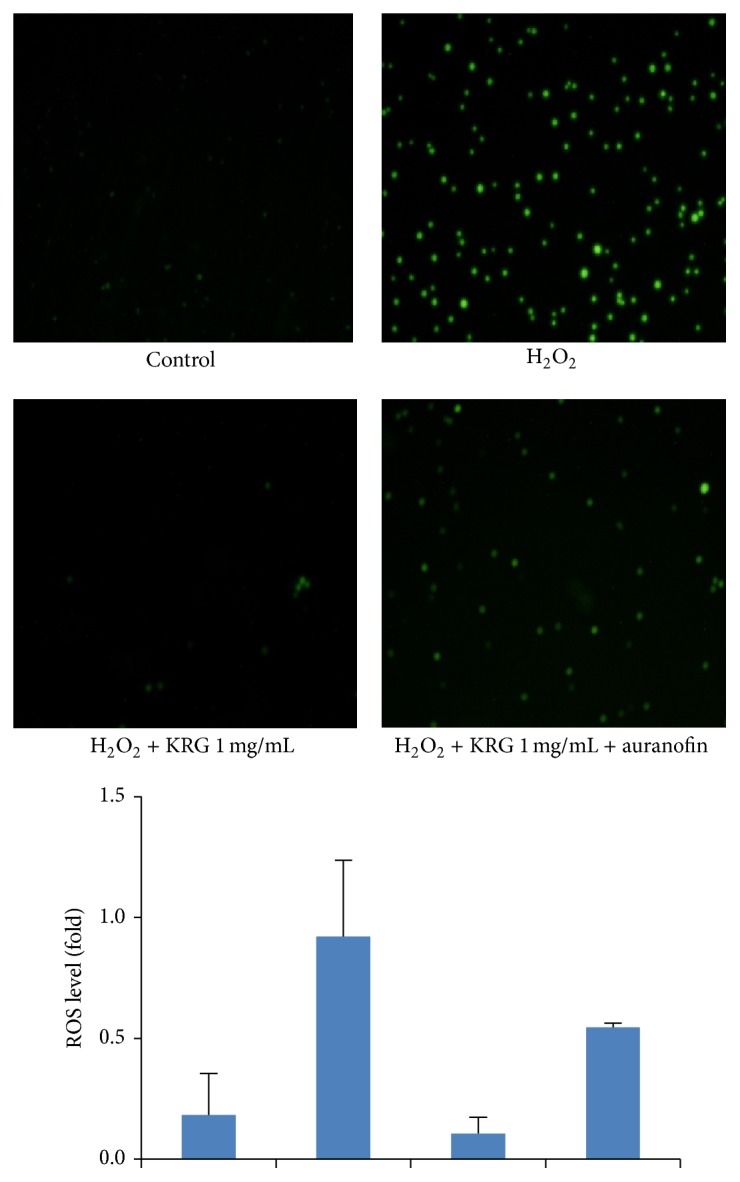
Intracellular reactive oxygen species level of KRG-stimulated HUVECs. HUVECs were pretreated for 1 h with or without 2 *μ*M auranofin and then incubated with 1 mg/mL KRG. After 18 h of incubation, HUVECs were stimulated with DCF/DA for 55 min, followed by treatment with 500 *μ*M H_2_O_2_ for 5 min. The protective effect of KRG on H_2_O_2_-induced cell death and the abrogation of this effect by the TrxR1 inhibitor, auranofin, were visualized by fluorescence microscopy.

ROS is an oxygen free radical that causes cellular damage. In endothelial cells, ROS aggravates oxidative injury and dysfunction, leading to cell death. To elucidate the function of TrxR1 as a ROS scavenger in KRG-treated HUVECs, we measured intracellular ROS levels using DCF/DA staining. While the intracellular ROS level increased when cells were stimulated with H_2_O_2_, ROS generation decreased after treatment with KRG and auranofin, a specific TrxR1 inhibitor (Figure 4). This indicates that upregulation of TrxR1 by KRG inhibits ROS production and prevents ROS-mediated cell damage and cell death.

### 3.5. Pharmacological Inhibition of TrxR1 Leads to Cell Death in KRG-Stimulated HUVECs

**Figure 5:**
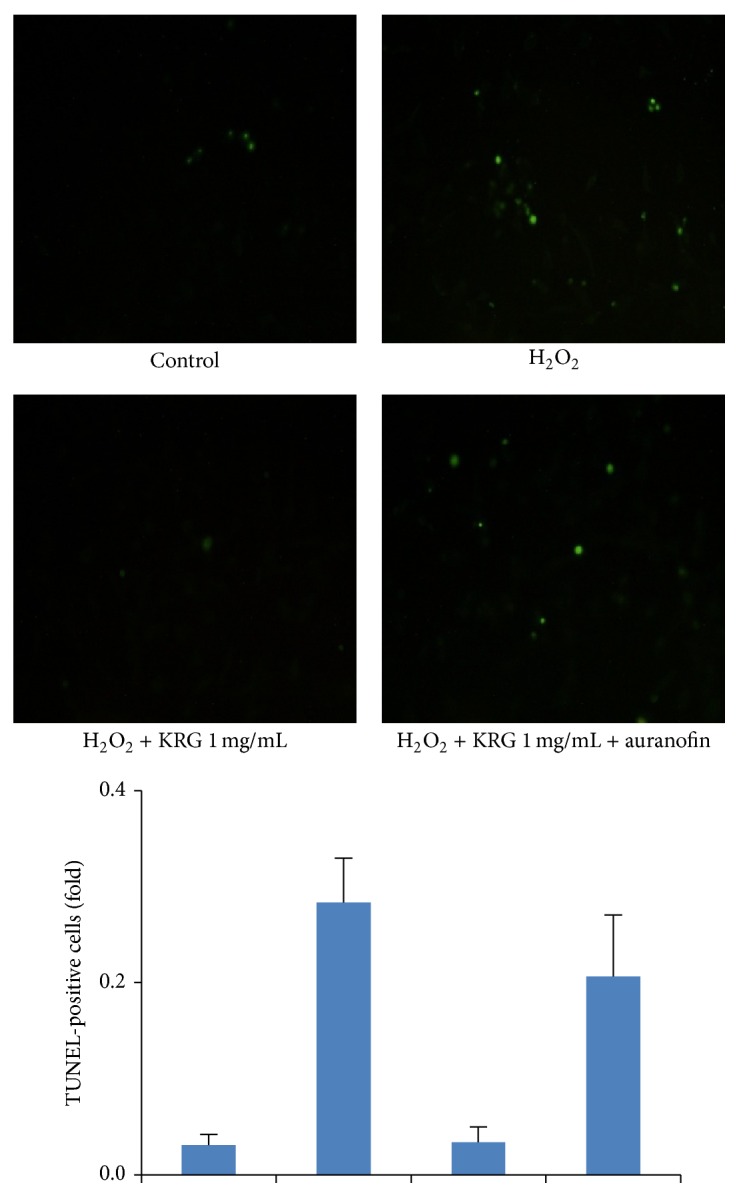
Terminal transferase dUTP nick end labeling (TUNEL) assay of TrxR1 inhibition in KRG-stimulated HUVECs. KRG 1 mg/mL treated HUVECs were pretreated for 1 h with or without 2 *μ*M auranofin, followed 1 h by treatment with 100 *μ*M H_2_O_2_. After 18 h of incubation, the protective effect of KRG on H_2_O_2_-induced cell death in HUVECs and its blockage by the TrxR1 inhibitor, auranofin, were determined by the TUNEL assay DAPI, 4′,6-diamidino-2-phenylindole.

Endothelium plays an important role of homeostasis and its dysfunction causes several physiological disorders such as atherosclerosis, diabetes, and hypertension. An accumulation of evidence has suggested that blockade of ROS production prevents endothelial dysfunction and reduces vascular diseases such as atherosclerosis. To further examine whether the upregulation of TrxR1 by KRG is responsible for the cytoprotective effect under oxidative stress, we suppressed the enzymatic activity of TrxR1 with the specific TrxR1 inhibitor, auranofin. KRG-treated cells were preincubated with or without 2 *μ*M auranofin, and the amount of cell death, including apoptosis, was analyzed by an* in situ* TUNEL assay that recognizes fragmented DNA. The results after KRG treatment were not significantly different from control cells. However, auranofin pretreatment decreased TrxR1 expression and increased the proportion of TUNEL-positive cells (Figure 5), indicating that the endothelial protective effect of KRG is related to TrxR1 expression and may prevent vascular disorders.

## 4. Discussion

In this study, we have confirmed that KRG increases the expression of the antioxidant enzyme, TrxR1, in KRG-stimulated human endothelial cells. We also show the induction of TrxR1 mRNA and protein expression levels, the signaling pathway of TrxR1 expression, and the antiapoptosis effect of TrxR1, all of which have cytoprotective properties in KRG-treated HUVECs.

Ginseng has many biological components including acidic polysaccharides, phenolic compounds, polyacetylenes, and ginsenosides. Because of its beneficial effects, ginseng has been used in Asia for more than 2,000 years as a therapy for various diseases [35]. When fresh ginseng is steamed between 90°C and 100°C and dried under sunlight, Red Ginseng is produced. High temperature and steam transform some of the components and, by suppressing catabolic enzymes, can enhance the pharmacological activity including the anti-inflammatory, anticarcinogenic, and antioxidant effects [35, 36]. The presence of the major ginsenosides such as Rg1 and Rb1 has been confirmed in KRG water extract [37]. KRG has been shown to decrease oxidative levels and protect endothelium by increasing nitric oxide (NO) production and eNOS and suppressing H_2_O_2_- or homocysteine-induced endothelial dysfunction [13, 38, 39]. In addition, KRG inhibits apoptosis via the mitochondrial caspase pathway, which prevents endothelium dysfunction and vascular diseases such as vascular inflammation and atherosclerosis [14, 40].

TrxR1 has homocysteine residues and reduces Trx and other small molecules that have cysteine residues. TrxR1 catalyzes the oxidized redox protein Trx by NADPH [41]. TrxR1 and Trx have a key role in maintaining redox homeostasis against oxidative stress produced by ROS [42]. Induction of TrxR1 and Trx protects oxidative stress-induced cell dysfunction and cell death through the regulation of transcription factors related to redox [43]. Numerous studies have found that Trx and TrxR1 expression can be used as markers of oxidative stress in biological fluids and tissues [23, 28, 44] and a study of oxidative stress showed that overexpressed Trx and TrxR1 act as intracellular H_2_O_2_-scavenging enzymes [45]. In addition, TrxR regenerates small antioxidant molecules including vitamin C or E and reduces the generation of lipid hydroperoxide in endothelial cells [41]. Altogether, the beneficial effects of TrxR1 mediate Trx systems that exert vascular protective effects by preventing oxidative stress and promoting endothelial cell survival [45, 46].

Studies have revealed that both KRG and TrxR1 have many beneficial effects. Previous evidence has showed that the expression of TrxR1 is induced by molecules such as flavonoids and teaflavin. Sugahara et al. [47] reported that one of the flavonoids, kaempferol, increased TrxR1 expression in human keratinocytes and exerted protective effects against oxidative stress. In addition, Yang et al. [11] showed that KRG induced various antioxidant enzymes, such as heme oxygenase-1 (HO-1), via the Nrf2 and ARE pathway in endothelial cells. However, there have been no reports on the relationship of KRG and TrxR1 or on the mechanism underlying the KRG-induced increase in TrxR1 in human endothelial cells. In this study, we have demonstrated that KRG induces TrxR1 expression via the p38 and PKC-*δ* signaling pathways. In addition, the induction of TrxR1 was shown to suppress the generation of intracellular ROS as well as apoptosis in KRG-stimulated HUVECs. A reduction in the levels of ROS generation induced by KRG promotes cell survival, which prevents endothelial dysfunction. Therefore, the increase in the expression of TrxR1 following treatment with KRG water extract may be associated with protective effects in the endothelium.

In this study, we suggest that KRG water extract may have a cytoprotective effect in endothelial cells via upregulation of TrxR1 expression through p38 and PKC-*δ* signaling. This study may provide a therapeutic mechanism for the use of KRG in various vascular diseases.
